# Trojan‐Horse‐Like Stimuli‐Responsive Microcapsules

**DOI:** 10.1002/advs.201700960

**Published:** 2018-03-13

**Authors:** Chuan‐Lin Mou, Wei Wang, Zhi‐Lu Li, Xiao‐Jie Ju, Rui Xie, Nan‐Nan Deng, Jie Wei, Zhuang Liu, Liang‐Yin Chu

**Affiliations:** ^1^ School of Chemical Engineering Sichuan University Chengdu Sichuan 610065 China; ^2^ College of Chemistry and Chemical Engineering Oil & Gas Field Applied Chemistry Key Laboratory of Sichuan Province Southwest Petroleum University Chengdu Sichuan 610500 China; ^3^ State Key Laboratory of Polymer Materials Engineering Sichuan University Chengdu Sichuan 610065 China

**Keywords:** interfaces, microfluidics, programmed release, stimuli‐responsive materials, Trojan‐horse‐like microcapsules

## Abstract

Multicompartment microcapsules, with each compartment protected by a distinct stimuli‐responsive shell for versatile controlled release, are highly desired for developing new‐generation microcarriers. Although many multicompartmental microcapsules have been created, most cannot combine different release styles to achieve flexible programmed sequential release. Here, one‐step template synthesis of controllable Trojan‐horse‐like stimuli‐responsive microcapsules is reported with capsule‐in‐capsule structures from microfluidic quadruple emulsions for diverse programmed sequential release. The nested inner and outer capsule compartments can separately encapsulate different contents, while their two stimuli‐responsive hydrogel shells can individually control the content release from each capsule compartment for versatile sequential release. This is demonstrated by using three types of Trojan‐horse‐like stimuli‐responsive microcapsules, with different combinations of release styles for flexible programmed sequential release. The proposed microcapsules provide novel advanced candidates for developing new‐generation microcarriers for diverse, efficient applications.

## 1 Introduction

Stimuli‐responsive microcapsules that enable on‐demand content release show great power for myriad applications such as drug delivery, self‐healing, and confined microreaction.^1^, ^2^, ^3^, ^4^, ^5^, ^6^, ^7^, ^8^, ^9^, ^10^, ^11^ For developing new‐generation microcarriers,^12^ multicompartmental microcapsules, with each compartment protected by a distinct and stable stimuli‐responsive shell for versatile coencapsulation and controlled release, are highly desired. Their multicompartments allow separate coencapsulation of multiple contents without crosscontamination, which is crucial to achieve enhanced performances for biomedical applications such as combination cancer therapy,^2^, ^13^, ^14^ tissue regeneration,^14^ theranostics,^15^ and confined enzymatic reactions.^16^ For example, in combination cancer therapy, codelivery of two or more drugs that work synergistically, such as drugs and genes,^13^ can achieve greater synergistic efficacy than the sum of each drug delivered alone. In tissue regeneration, codelivery of two different growth factors allows formation of mature vessels while delivery of one alone is insufficient for the promotion of a stable, dense vasculature.^14^ In confined enzymatic microreactions, coencapsulation of multiple enzymes in the separate compartments of a microcarrier can provide improved enzymatic activity and stability as compared with free enzymes and enzymes homogeneously coimmobilized in a microcarrier.^16^ Based on the coencapsulation, flexible control of the release sequence of different contents can further achieve therapeutic synergy for enhanced chemotherapy and reduced toxicity.^17^, ^18^, ^19^, ^20^ For example, sequential release of multiple therapeutic agents allows first genetically sensitizing the cancer cells to the sequentially administered drug for enhanced chemotherapy.^17^ Improved therapeutic index with reduced toxicity can also be realized via sequential release of an antiangiogenesis agent for vascular collapse, and then a cytotoxic agent for chemotherapy inside a tumor.^19^ Moreover, besides the coencapsulation and sequential release, control of the compartment structure, capsule size, and uniformity allows accurate adjustment of the stoichiometric ratio and release kinetics of the contents for optimized efficacy.^21^, ^22^, ^23^ However, although many multicompartmental microcapsules have been developed,^1^, ^3^, ^4^, ^5^, ^12^, ^16^, ^21^, ^24^, ^25^, ^26^, ^27^, ^28^, ^29^, ^30^, ^31^, ^32^, ^33^, ^34^, ^35^ most cannot combine different release styles to achieve flexible programmed sequential release.

Usually, multicompartmental microcapsules, with nested polymersomes,^6^, ^26^, ^31^ liposomes,^28^ or polyelectrolytes structures,^3^, ^4^ can be fabricated by multistep self‐assembly, and particle‐template assembly. However, they require troublesome multistep process and show uncontrollable multicompartments due to the limited control of capsule size and inner capsule number. With microfluidics for precise manipulation of microdrops,^36^, ^37^, ^38^, ^39^ uniform multiple emulsions can be generated for one‐step template synthesis of multicompartmental microcapsules with versatile compartment structures and controllable sizes.^1^, ^24^, ^29^, ^32^, ^33^, ^34^, ^39^, ^40^ By using double or triple emulsions as templates, microcapsules with a shell of hydrogel,^39^, ^40^ lipid,^24^, ^34^ or assembled copolymers,^29^, ^32^ containing parallel core compartments or more hierarchical multicompartments, can be fabricated. However, these microcapsules with one single shell only can corelease their contents together upon one triggering. By loading preformed polymersomes into the inner drop of double emulsions, microcapsules with nested polymersomes structures can be fabricated for sequential release via selective shell dissolution by ethanol–water mixture.^33^ However, precise control of their compartmental structures still remains difficult due to the randomly loaded inner polymersomes in the emulsion templates. Moreover, for the assembled copolymer shells, their poor stability against osmotic pressure or mechanical stress usually causes undesired shell rupture and content release.^29^ Recently, from Pickering emulsion drops, microcapsules, containing a more stable pH‐responsive hydrogel shell, decorated with porous silica nanoparticles modified by thermo‐responsive polymers, have been created for programmed sequential release.^25^ Nevertheless, the microcapsules show nonuniform size and limited control of loading amount of contents in the silica nanoparticles due to limited shell surface. Moreover, complete versatility of multicompartmental microcapsules still requires flexible combination of different functional shells for diverse programmed sequential release. Thus, creation of uniform controllable multicompartmental microcapsules, with each compartment protected by a distinct and stable stimuli‐responsive shell for flexible programmed sequential release, is still highly desired.

**Figure 1:**
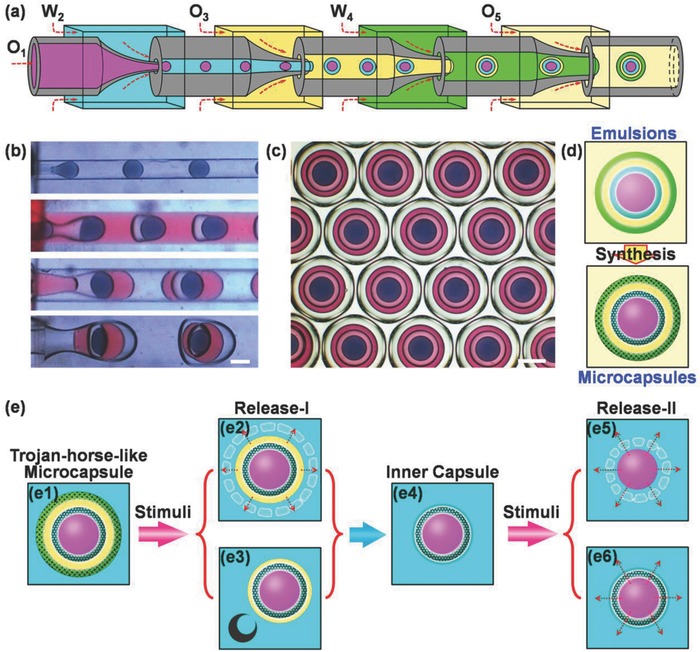
Schematic illustration showing the strategy for template synthesis of Trojan‐horse‐like microcapsules with capsule‐in‐capsule structures for programmed sequential release. a–d) Glass‐capillary microfluidic device a) for generating uniform O_1_/W_2_/O_3_/W_4_/O_5_ quadruple emulsions c) via sequential emulsifications b) for template synthesis of Trojan‐horse‐like microcapsules d). e) The microcapsules e1) for programmed two‐stage sequential release via stimuli‐triggers. Stage I: Outer capsule for burst release of outer oil core (O_3_) and inner capsule via stimuli‐triggered shell decomposition e2) or shell shrinking/rupturing e3); Stage II: inner capsule e4) for burst release of inner oil core (O_1_) via stimuli‐triggered shell decomposition e5) or diffusion‐based sustained release e6). Scale bars are 200 µm.

Here, we report on a facile and flexible strategy for controllable one‐step fabrication of uniform Trojan‐horse‐like stimuli‐responsive microcapsules with capsule‐in‐capsule structures for diverse programmed sequential release. The microcapsules contain two stimuli‐responsive hydrogel shells to individually control the content release from each capsule compartment. Uniform O_1_/W_2_/O_3_/W_4_/O_5_ quadruple emulsions generated from microfluidics are used as templates (**Figure** **1**a–c). The composition of each phase in the multilayered emulsion templates is elaborately engineered to provide stable interfaces for microcapsule synthesis. The quadruple emulsions are converted to multicompartmental microcapsules with capsule‐in‐capsule structures, by adding different functional shell materials into their inner (W_2_) and outer (W_4_) aqueous layers (Figure 1d). This allows flexible engineering of different triggering mechanisms into the two shells for versatile programmed sequential release. To demonstrate this, three types of Trojan‐horse‐like stimuli‐responsive microcapsules, with flexible combinations of stimuli‐responsive shells, are created for programmed sequential release. The microcapsules enable burst release via shell decomposition (Figure 1e1,e2) or shell rupture (Figure 1e1,e3) for the first release, followed with burst release via shell decomposition (Figure 1e4,e5) or sustained release (Figure 1e4,e6) for the second release. The proposed Trojan‐horse‐like microcapsules provide novel advanced candidates as codelivery microcarriers for programed sequential release, and as microreactors for triggered microreactions.

## 2 Results and Discussion

### 2.1 Microfluidic Generation of Monodisperse Quadruple Emulsion Templates

O_1_/W_2_/O_3_/W_4_/O_5_ quadruple emulsions generated from a glass‐capillary microfluidic device are utilized as templates for synthesis of Trojan‐horse‐like microcapsules with capsule‐in‐capsule structures (Figure 1). The capillary microfluidic device is fabricated by assembly of cylindrical and square glass capillaries on glass plates according to our previous work.^39^ For generating the quadruple emulsions, briefly, the aqueous phases W_2_ and W_4_ are deionized water with surfactant 0.5% (w/v) Pluronic F‐127 and 10% (w/v) glycerin; the oil phases O_1_ and O_3_ are the mixture of soybean oil (SO) and benzyl benzoate (BB) with *V*_SO_:*V*_BB_ = 46:54, containing 2% (w/v) and 4% (w/v) surfactant polyglycerol polyricinoleate (PGPR), respectively; O_5_ and collection solution are SO containing 5% (w/v) PGPR. The density of the O_1_ and O_3_ (1.025 g mL^−1^) is adjusted as the same as that of the aqueous phases (1.024 g mL^−1^). To better observe the nested drop‐in‐drop structures of the quadruple emulsions, oil‐soluble dyes Sudan Black and Lumogen^@^ F Red 300 (LR 300) are added in the O_1_ and O_3_ phases (see Table S1 in the Supporting Information for recipe, density, and viscosity of the emulsions). After injecting the oil and water phases into the microfluidic device at appropriate flow rates via pumps, O_1_/W_2_/O_3_/W_4_/O_5_ quadruple emulsions can be produced via sequential emulsification in microchannels (Figure 1b and Movie S1, Supporting Information). The produced quadruple emulsions, collected in the collection solution, show uniform and stable nested drop‐in‐drop structures (Figure 1c). The values of coefficient of variation (CV), which is defined as the ratio of the standard deviation of size distribution to its arithmetic mean, are all less than 3% for drops of each phase in the quadruple emulsions, indicating high monodispersity. The multilayered quadruple emulsions provide excellent templates for fabrication of multicompartment microcapsules with capsule‐in‐capsule structures.

### 2.2 Fabrication of Trojan‐Horse‐Like Chitosan@Chitosan Microcapsules

Trojan‐horse‐like microcapsules with controllable capsule‐in‐capsule structures are fabricated by selectively incorporating functional shell materials in the separate W_2_ and W_4_ phases of quadruple emulsions for microcapsule fabrication. First, we show the fabrication of Trojan‐horse‐like microcapsules with two shells of same composition, by incorporating chitosan into the W_2_ and W_4_ phases. After generation of quadruple emulsions, the terephthalaldehyde (TA) added in the oil phases can diffuse across the W/O interfaces into W_2_ and W_4_ phases to crosslink the chitosan. This can convert the W_2_ and W_4_ phases into chitosan hydrogel shells to create multicompartmental microcapsules with capsule‐in‐capsule structures (CS@CS microcapsules). Since this is a typical process that involves mass transfer across the interfaces and interfacial crosslinking reaction, the interface stability of the quadruple emulsions under such interfacial mass transfer and reaction is crucial for efficient conversion of the emulsion templates into microcapsules.

**Figure 2:**
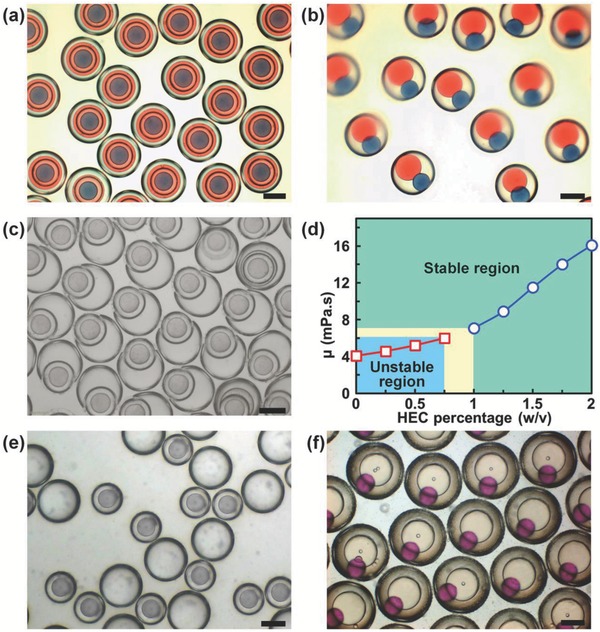
Evolution of O_1_/W_2_/O_3_/W_4_/O_5_ quadruple emulsions during the microcapsule synthesis. a,b) Optical microscopy images of quadruple emulsions with W_2_ a,b) and W_4_ c) of lower viscosity (4.1 mPa·s) before a) and after b,c) rupture during the synthesis of CS@CS microcapsule. d) Effect of viscosity of aqueous phase on CS@CS microcapsule synthesis. e) Optical microscopy image of ruptured quadruple emulsions with O_3_ of lower density (0.917 g mL^−1^) during the synthesis of CS@CS microcapsule. f) Optical microscopy image of ruptured quadruple emulsions with W_2_ of lower viscosity (4.1 mPa·s) during the synthesis of CS@PNIPAM microcapsule. Scale bars are 200 µm.

Effects of the interfacial mass transfer and reaction on the interface stability of emulsion templates are studied to optimize the recipes for efficient microcapsule synthesis (see Supporting Information for detailed discussion, and Tables S1–S12 in the Supporting Information for recipe, density, and viscosity of the emulsions). Quadruple emulsions without the shell materials are used as control group (See Tables S1 and S2 in the Supporting Information for recipe, density, and viscosity of the emulsions). First, the interface stability of quadruple emulsions with chitosan‐containing aqueous phases of different viscosities is studied during the synthesis process of CS@CS microcapsules. Typically, the surfactant contents are fixed to keep fixed interfacial tensions (≈0.11 mN m^−1^) between aqueous and oil phases, and the densities of the dispersed phases are nearly the same (≈1.0 g mL^−1^). Deionized water, with pH adjusted to 6.6, containing 0.5% (w/v) Pluronic F127 and 4% (w/v) chitosan for shell construction (marked as W_CS−1_) is used as W_2_ phase (μ = 4.1 mPa·s), while W_CS−1_ added with 1.5% (w/v) hydroxyethyl cellulose (HEC) for viscosity adjustment (W_CS−2_) is used as W_4_ phase (μ = 11.5 mPa·s). Meanwhile, 2% (w/v), 0.2% (w/v), and 0.2% (w/v) TA are, respectively, added in the original O_1_ and O_3_ phases, and the collection solutions (see Table S3 for the recipe, density, and viscosity of the emulsions, and Table S4 for the interfacial tensions in the Supporting Information). The collected quadruple emulsions initially show uniform nested drop‐in‐drop structures (**Figure** **2**a), but the interfaces of W_2_ phase break after 10 min due to its lower viscosity. This leads to transition of the quadruple emulsions into double emulsions containing oil drops of O_1_ and O_3_ (Figure 2b). Contrarily, when W_CS−1_ and W_CS−2_ are, respectively, used as W_4_ and W_2_ phases (see Table S5 for the recipe, density, and viscosity of the emulsions, and Table S6 for the interfacial tensions in the Supporting Information), the uniform quadruple emulsions (Figure S1, Supporting Information) break after collected for 10 min (Figure 2c), due to the interface break of less viscous W_4_ phase. Especially, W_2_ and W_4_ phases with μ ≥ 7.1 mPa·s allow stable interfaces for efficient conversion of the emulsions to microcapsules, while those with μ < 6.0 mPa·s lead to failed conversion (Figure 2d). Second, the interface stability of quadruple emulsions with density difference between the oil and chitosan‐containing aqueous phases is studied during the synthesis process of CS@CS microcapsules. Typically, the interfacial tensions between aqueous and oil phases are also kept nearly the same (≈0.11 mN m^−1^). The W_CS−2_ (μ = 11.5 mPa·s), with density (ρ = 1.013 g mL^−1^) similar to that of the O_1_ phase (ρ = 1.025 g mL^−1^), is used as W_2_ and W_4_ phases to provide stable interfaces. Meanwhile, SO with PGPR (4%, w/v) and TA (0.2%, w/v) is used as O_3_ phase (ρ = 0.917 g mL^−1^) to create the density difference (see Table S7 for the recipe, density, and viscosity of the emulsions, and Table S8 for the interfacial tensions in the Supporting Information). Due to the density mismatch between O_3_ and aqueous phases, the interfaces of O_3_ phase rupture to break each of the quadruple emulsions (Figure S2, Supporting Information) into an O/W/O emulsion drop and a W/O emulsion drop (Figure 2e) during microcapsule synthesis. However, it is worth noting that without the interfacial mass transfer and reaction, the quadruple emulsions with aqueous phase of much lower viscosity (Tables S1 and S2, Supporting Information), and larger density differences (Table S2, Supporting Information) between the aqueous and oil phases can remain stable. The results confirm the effects of interfacial mass transfer and reaction on the interface stability of quadruple emulsions during the microcapsule synthesis.

**Figure 3:**
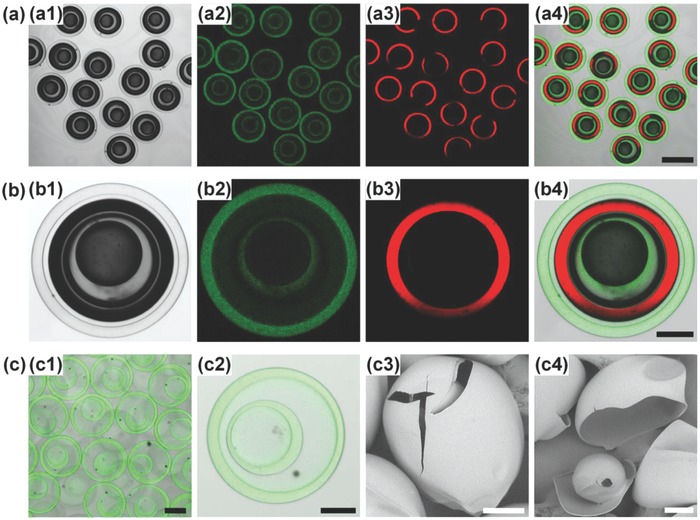
Trojan‐horse‐like CS@CS microcapsules. a,b) Optical a1,b1) and CLSM a2–a4,b2–b4) images of CS@CS microcapsules a) and single CS@CS microcapsule b). The chitosan shell shows green fluorescence, while oil phase with LR300 shows red fluorescence. c) CLSM images of CS@CS microcapsules after washing away the inner oil phases c1,c2), and their SEM images before c3) and after c4) cracking. Scale bars are 400 µm in (a), 200 µm in (c1), and 100 µm in (b, c2–c4).

**Figure 4:**
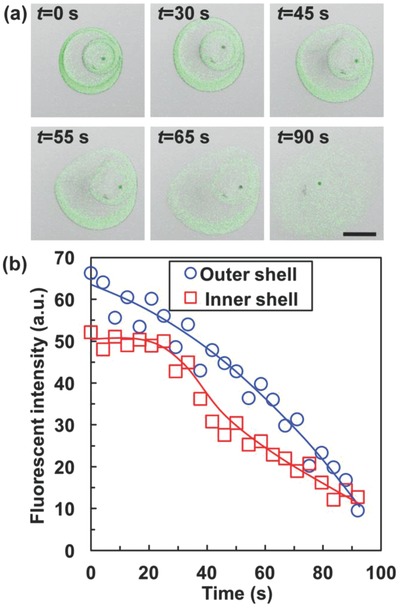
Acid‐triggered shell decomposition of CS@CS microcapsules. a) CLSM images showing the shell decomposition behavior of CS@CS microcapsules upon addition of buffer solution (pH = 2.5). b) Time‐dependent change of fluorescent intensities of the two chitosan shells in CS@CS microcapsules. Scale bars are 200 µm.

Based on the results, with optimized recipe (Table S9, Supporting Information) for matched viscosity and density to provide stable interfaces, the quadruple emulsions can be efficiently converted into CS@CS microcapsules. The produced CS@CS microcapsules show uniform capsule‐in‐capsule structures (**Figure** **3**a), with average size of ≈419 µm. The CV values of the inner and outer capsules are lower than 4%, indicating an efficient conversion process. Since TA‐crosslinked chitosan shows autofluorescent properties due to the formation of Schiff's bases,^41^ the confocal laser scanning microscopy (CLSM) images of microcapsules clearly show the two chitosan shells (green fluorescence) (Figure 3b,c1,c2). Meanwhile, O_1_ and O_3_ cores with different contents can be separately loaded in the inner and outer capsule compartments (Figure 3b) for coencapsulation. Moreover, the scanning electron microscopy (SEM) images of CS@CS microcapsules before (Figure 3c3) and after (Figure 3c4) cracking further confirm the presence of a smaller capsule inside the outer shell. The chitosan shells, which are crosslinked by TA via formation of Schiff's bases, can show good stability in water against degradation. Since the Schiff's bases are prone to degradation at low pH, decomposition of the chitosan shells can be achieved by adding acid. **Figure** **4**a shows the decomposition process of the two chitosan shells upon recognizing decrease in pH value (pH = 2.5). During the acid‐triggered shell decomposition process, the acid first contacts with the outer shell for shell decomposition, resulting in decreased fluorescent intensity (Figure 4b). This process leads to delayed acid diffusion to the inner shell and consumption of the acid. Thus, protected by the outer shell against fast decomposition at the beginning, the fluorescent intensity of inner shell remains nearly unchanged for the first ≈30 s, and then decreases (Figure 4b). This sequential shell decomposition process can benefit the sequential release of different contents from the outer and inner capsules.

### 2.3 Fabrication of Trojan‐Horse‐Like Poly(Ethylene Glycol) Diacrylate@Chitosan and Chitosan@Poly(*N*‐Isopropylacrylamide) Microcapsules

**Figure 5:**
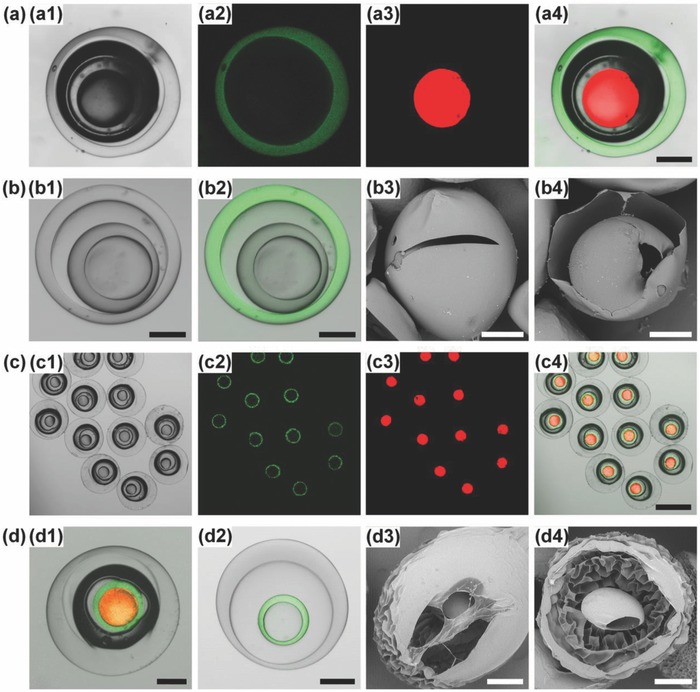
Trojan‐horse‐like PEGDA@CS microcapsules and CS@PNIPAM microcapsules. a) Optical a1) and CLSM images a2–a4) of PEGDA@CS microcapsules. b) Optical b1) and CLSM b2) images of single PEGDA@CS microcapsule after washing away the inner oil phases, and their SEM images before b3) and after b4) cracking. c) Optical c1) and CLSM images c2–c4) of CS@PNIPAM microcapsules. d) CLSM images of single CS@PNIPAM microcapsule before d1) and after d2) washing away the inner oil phases, and their SEM images before d3) and after d4) cracking. The chitosan shell shows green fluorescence, while oil phase with LR300 shows red fluorescence. Scale bars are 100 µm in (a,b,d) and 400 µm in (c).

Trojan‐horse‐like microcapsules with two distinct shells can be fabricated by incorporating two different shell materials into the aqueous phases. This is first demonstrated by respectively incorporating poly(ethylene glycol) diacrylate (PEGDA) and chitosan into W_2_ and W_4_ to engineer inner PEGDA shell and outer chitosan shell (PEGDA@CS microcapsules) (Table S10, Supporting Information). Briefly, deionized water with 0.5% (w/v) Pluronic F127, 10% (w/v) PEGDA, and 0.5% (w/v) photo‐initiator 2,2′‐azobis(2‐methylpropionamidine) dihydrochloride (V‐50) is used as W_2_ phase for constructing the inner PEGDA shell, while the W_CS−2_ is used as W_4_ phase for constructing the outer chitosan shell. The CLSM images of PEGDA@CS microcapsules clearly show their capsule‐in‐capsule structures, with a nonfluorescent inner PEGDA shell and a fluorescent outer chitosan shell (**Figure** **5**a,b1,b2) for separately encapsulating oil cores with different contents (Figure 5a). The average size of these PEGDA@CS microcapsules is ≈424 µm. Meanwhile, the SEM images (Figure 5b3,b4) also confirm their capsule‐in‐capsule structures.

**Figure 6:**
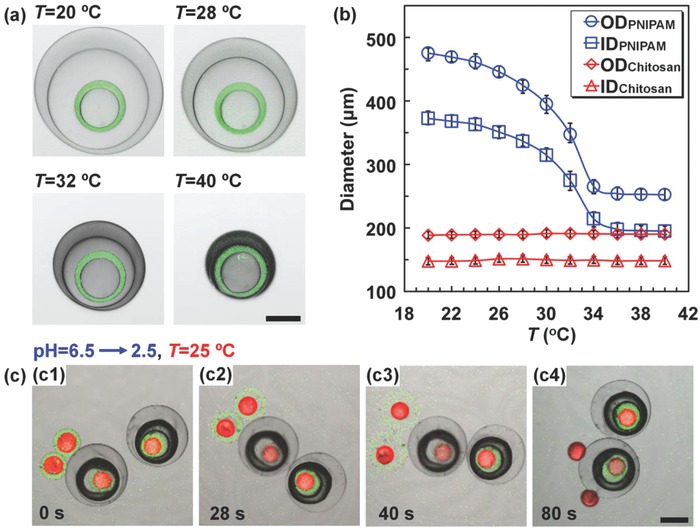
Trojan‐horse‐like CS@PNIPAM microcapsules with thermo‐responsive outer shell for inner capsule protection. a) CLSM images showing the temperature‐triggered shrinking of their outer PNIPAM shell. b) Temperature‐dependent change of inner (ID) and outer (OD) diameters of the inner chitosan and outer PNIPAM shells. c) Effect of acidic environment on the shell decomposition of core–shell chitosan microcapsules and inner chitosan capsules protected in CS@PNIPAM microcapsules. The chitosan shell shows green fluorescence, while oil phase with LR300 shows red fluorescence. Scale bars are 100 µm in (a) and 200 µm in (c).

Next, we demonstrate the flexibility of our strategy for fabricating the microcapsules with two distinct shells by respectively incorporating chitosan and poly(*N*‐isopropylacrylamide) (PNIPAM) into W_2_ and W_4_ phases. This can produce Trojan‐horse‐like microcapsules with inner chitosan shell and outer PNIPAM shell (CS@PNIPAM microcapsules). During the microcapsule synthesis, the interfacial mass transfer and reaction involved in the fabrication of the inner chitosan shell also influence the interfacial stability. Typically, deionized water with monomer *N*‐isopropylacrylamide (NIPAM) (11.3%, w/v), crosslinker *N*,*N*′‐methylene bisacrylamide (0.77%, w/v), V‐50 (0.5%, w/v), Pluronic F‐127 (0.5%, w/v), and glycerol (5%, w/v) is used as W_4_ phase for constructing the outer PNIPAM shell. SO containing PGPR (4%, w/v) and TA (0.2%, w/v) is used as O_3_ phase (ρ = 0.917 g mL^−1^) to create density difference from the aqueous phases (W_2_ and W_4_). When W_CS−1_ is used as W_2_ (μ = 4.1 mPa·s) for constructing the inner chitosan shell, the quadruple emulsions transform to double emulsions (Figure S3, Supporting Information) containing drops of O_1_ and O_3_ (Figure 2f); because the interfaces of the chitosan‐containing W_2_ phase breaks due to its lower viscosity and density difference from O_3_. However, although W_4_ phase shows a different density from O_3_ and a much lower viscosity (1.68 mPa·s) (see Table S11 in the Supporting Information for the recipe, density, and viscosity of the emulsions), the interfaces of the NIPAM‐containing W_4_ phase can remain stable during the shell synthesis, because the synthesis of PNIPAM shell based on UV polymerization only involves reaction inside the W_4_ phase, without interfacial mass transfer and interfacial reaction. The results further confirm the influences of interfacial mass transfer and reaction on the interface stability of quadruple emulsions during the microcapsule synthesis. Based on the results, with optimized composition containing W_CS−2_ as W_2_ phase to provide stable interfaces, uniform CS@PNIPAM microcapsules can be fabricated efficiently from quadruple emulsion templates (see Table S12 in the Supporting Information for the recipe, density, and viscosity of the emulsions). The nonfluorescent outer PNIPAM shell and fluorescent inner chitosan shell in CLSM images (Figure 5c,d1,d2), and the cracked microcapsule in SEM images (Figure 5d3,d4) both confirm the capsule‐in‐capsule structures of the resultant CS@PNIPAM microcapsules. The average size of these CS@PNIPAM microcapsules is ≈461 µm. With distinct inner and outer shells, different stimuli‐responsive properties can be integrated into one single microcapsule for flexible sequential release behaviors. As shown in **Figure** **6**a,b, when increasing the temperature across the volume phase transition temperature of PNIPAM (≈32 °C), the outer thermo‐responsive PNIPAM shell shrinks dramatically; meanwhile, the inner chitosan shell keeps an unchanged size, indicating their different functions. Such a feature can benefit the flexible combination of different triggering mechanisms in the Trojan‐horse‐like microcapsules for versatile sequential release. Moreover, as compared with core–shell chitosan microcapsules without outer shell protection, the PNIPAM shell and O_3_ phase of Trojan‐horse‐like CS@PNIPAM microcapsules can protect the inner chitosan capsule against fast acid‐induced decomposition (Figure 6c). During the process, the outer PNIPAM shell and O_3_ phase act as barriers to delay the diffusion of acid into the inner chitosan capsule. Thus, the inner chitosan capsules remain intact at the beginning. Driven by the concentration gradient, finally, the acid can diffuse through the outer PNIPAM shell and O_3_ phase, and then decompose the inner chitosan capsule.

### 2.4 Trojan‐Horse‐Like Microcapsules for Versatile Programmed Sequential Release

**Figure 7:**
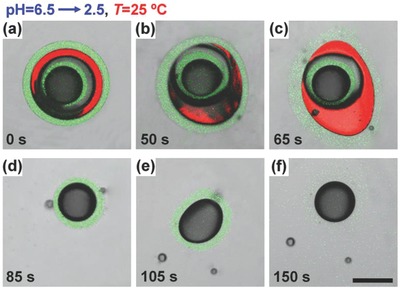
CS@CS microcapsules for programmed sequential release. CLSM images showing the acid‐triggered burst release of inner chitosan capsule a–d) for further acid‐triggered burst release e,f). Scale bar is 200 µm.

The Trojan‐horse‐like microcapsules with different combinations of functional shells enable flexible triggering mechanisms to achieve versatile programmed sequential release behaviors. This is demonstrated by first using the CS@CS microcapsules for acid‐triggered sequential release by adding buffer solution with pH = 2.5. Upon contacting with the acid solution, the outer chitosan shell of the microcapsules decomposes first and releases the LR300‐loaded O_3_ phase and inner chitosan capsule (**Figure** **7**a–d). Since the O_3_ phase delays the acid diffusion, the inner chitosan shell then decomposes to release the innermost Sudan‐Black‐loaded O_1_ phase (Figure 7e,f and Movie S2, Supporting Information).

**Figure 8:**
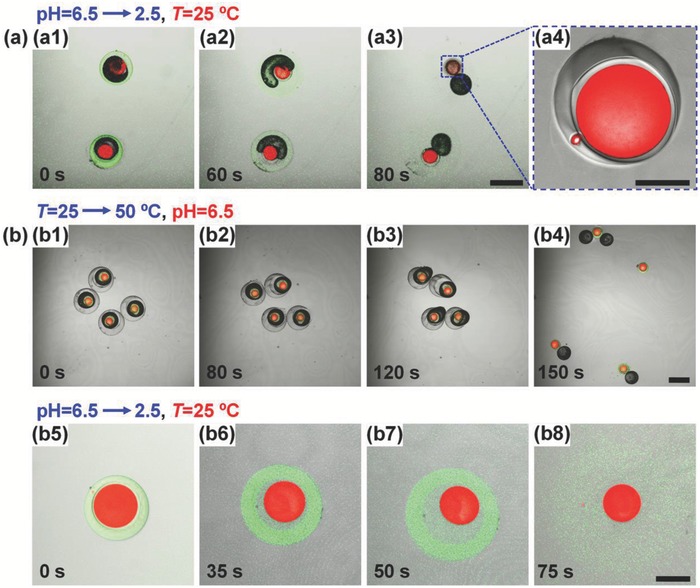
PEGDA@CS microcapsules and CS@PNIPAM microcapsules for programmed sequential release. a) CLSM images of PEGDA@CS microcapsules showing the acid‐triggered burst release a1–a3) of inner PEGDA capsule a4) for further sustained release. b) CLSM images of CS@PNIPAM microcapsules showing the thermo‐triggered burst release of inner chitosan capsule b1–b4) for further acid‐triggered burst release b5–b8). Scale bars are 400 µm in (a1–a3,b1–b4), and 100 µm in (a4,b5–b8).

As compared to the microcapsules with two shells of same composition, microcapsules with two distinct shells allow flexible combination of different release styles for more versatile programmed sequential release. As demonstrated here, typically, the PEGDA@CS microcapsules can achieve an acid‐triggered burst release at first stage, followed with a sustained release at the second stage. Upon decreasing pH to 2.5, the outer chitosan shell decomposes to release the middle O_3_ phase and inner PEGDA capsule (**Figure** **8**a1–a3). Since the PEGDA shell cannot be decomposed by acid, the released inner capsule remains intact (Figure 8a4) to allow further diffusion‐based sustained release of contents. Alternatively, for CS@PNIPAM microcapsules, sequential burst release can be achieved by first using a thermo‐trigger and then an acid‐trigger. Upon increasing temperature from 25 to 50 °C, the outer PNIPAM shell shrinks to squeeze the encapsulated oil phases and inner chitosan capsule. Due to the incompressible inner oil phases, the shrinking can lead to an increasing pressure on the PNIPAM shell. Finally, the PNIPAM shell ruptures and results in fast release of the encapsulated O_3_ phase and inner chitosan capsule into the environment (Figure 8b1–b4 and Movie S3, Supporting Information). Such a burst release can show a much faster spreading speed as compared to the release case driven by diffusion.^41^, ^42^ Then, for the second release, the deionized water is switched to buffer solution (pH = 2.5) at 25 °C to decompose the released chitosan capsule, thus leading to the release of the LR300‐loaded O_1_ core (Figure 8b5–b8). All the results show the flexible combinations of release styles in the Trojan‐horse‐like microcapsules for versatile programmed sequential release. Moreover, the release sequence of the contents in the outer and inner capsule compartments can be easily tuned by reversing the triggers. This can lead to contents mix of the inner O_1_ and O_3_ phases within the outer capsule, showing potential for confined microreactions.

## 3 Conclusions

In summary, controllable Trojan‐horse‐like stimuli‐responsive microcapsules with capsule‐in‐capsule structures for versatile programmed sequential release are developed by one‐step template synthesis from microfluidic quadruple emulsions. The emulsion templates can be converted into the microcapsules with hierarchical capsule‐in‐capsule structures, by incorporating different functional shell materials into their inner and outer aqueous layers. This also enables flexible engineering of different triggering mechanisms into the two shells to achieve versatile programmed sequential release. The microcapsules can controllably load different contents in their separate capsule compartments and release each of the contents in a predefined order and different manner. Further, control of the compartment size and shell thickness by tuning flow rates enables fine adjustment of the content amount in each compartment as well as their release profiles. Meanwhile, the outer and inner shells of microcapsules can be engineered with diverse stimuli‐responsive materials,^43^ such as ion‐responsive,^44^ glucose‐responsive,^45^ and multistimuli‐responsive ones,^46^ for more flexible codelivery. With controllable number and composition of inner drops,^38^, ^39^ the diverse multiple emulsions from microfluidics create opportunities to further fabricate Trojan‐horse‐like microcapsules containing inner capsules with controlled numbers and with different stimuli‐responsive shells for more versatile sequential release. Moreover, based on the microfluidic techniques for producing multiple emulsions,^47^, ^48^, ^49^ the microcapsule size can be further adjusted in the range from several tens of micrometers to a few millimeters. With such a size range, the microcapsules can be used for in vivo drug release via routes such as oral, subcutaneous, and intramuscular administrations.^50^, ^51^, ^52^ The proposed Trojan‐horse‐like stimuli‐responsive microcapsules provide novel advanced candidates for developing new‐generation microcarriers for programed sequential release, and on‐demand microreactions.

## 4 Experimental Section

*Microfluidic Generation of Quadruple Emulsions*: A glass‐capillary microfluidic device, constructed according to the previous work,^39^ was used for generating quadruple emulsions as templates for synthesis of the Trojan‐horse‐like microcapsules. For generating the O_1_/W_2_/O_3_/W_4_/O_5_ quadruple emulsions, typically, an oil mixture of SO(Kerry Oils & Grains Co., Ltd.) and BB(Sinopharm Chemical Reagent Co., Ltd.) with *V*_SO_:*V*_BB_ = 46:54, containing 2% (w/v) surfactant PGPR(Danisco), was used as the O_1_ phase. Deionized water (Milli‐Q) containing 0.5% (w/v) surfactant Pluronic F‐127 (Sigma‐Aldrich) and 10% (w/v) glycerin was used as the W_2_ and W_4_ phases. O_3_ phase was the SO–BB mixture containing PGPR (4%, w/v), while O_5_ phase and the collection solution were SO containing PGPR (5%, w/v) (Table S1). These solutions were injected into the microfluidic device by syringe pumps (LSP01‐1A, Baoding Longer Precision Pump) for emulsion generation. The flow rates of O_1_, W_2_, O_3_, W_4_, and O_5_ were 230, 440, 850, 1400, and 6000 µL h^−1^, respectively. Dyes Sudan Black (0.1%, w/v) and Lumogen@ F Red 300 (LR 300) (0.1%, w/v) were, respectively, added in O_1_ and O_3_ phases for better observation. For fabricating the Trojan‐horse‐like microcapsules, the W_2_ and W_4_ phases of the quadruple emulsions were added with functional shell materials.

*Characterization of Emulsions*: The formation process of quadruple emulsions was monitored by using a high‐speed camera (Phantom Miro 3, Vision Research). The morphology and stability of the quadruple emulsions were investigated by using an inverted optical microscope (IX71, Olympus) equipped with a charge‐coupled‐device (CCD) camera. The size and size distribution of the quadruple emulsions were determined from their optical microscopy images by using an automatic analytic software. To quantify the monodispersity of the quadruple emulsions and the inner drops, the sizes of 100 emulsion drops were measured to calculate their CV (defined as the ratio of the standard deviation of the size distribution to its arithmetic mean). The viscosity of the aqueous and oil phases was measured by using viscosimeter (Brookfield DV2T). The interfacial tensions between the aqueous and oil phases were measured by using a drop shape analyzer (DSA25, Krüss GmbH).

*Fabrication of Trojan‐Horse‐Like CS@CS Microcapsules*: For fabricating Trojan‐horse‐like microcapsules with two chitosan shells, deionized water containing 0.5% (w/v) Pluronic F‐127, 4% (w/v) chitosan (*M*_w_ = 5000, degree of deacetylation was 85%, Ji'nan Haidebei Marine Bioengineering Co., Ltd.) for shell construction, and 1.5% (w/v) HEC for viscosity adjustment was used as W_2_ and W_4_ phases. The pH value of all chitosan‐containing aqueous phases in the experiments was adjusted to 6.6 by dropwise adding 1.0 mol L^−1^ NaOH. TA was added in the original O_1_ and O_3_ phases with contents of 2% (w/v) and 0.2% (w/v), respectively, and in the collection solution with content of 0.2% (w/v) for crosslinking the chitosan. Moreover, Sudan Black (0.1%, w/v) and LR 300 (0.1%, w/v) were, respectively, added as model chemical molecules in O_1_ and O_3_ phases for release (Table S9, Supporting Information). The flow rates of O_1_, W_2_, O_3_, W_4_, and O_5_ for emulsion generation were 220, 460, 900, 2200, and 8000 µL h^−1^, respectively. After collected in the collection solution at room temperature for 12 h, the quadruple emulsions were converted into CS@CS microcapsules due to the diffusion of TA from oil phases to the aqueous phases for chitosan crosslinking.

*Fabrication of Trojan‐Horse‐Like PEGDA@CS Microcapsules*: For fabricating Trojan‐horse‐like PEGDA@CS microcapsules, deionized water containing 0.5% (w/v) Pluronic F‐127, 10% (w/v) PEGDA (Sigma‐Aldrich), and 0.5% (w/v) photo‐initiator 2,2′‐azobis(2‐methylpropionamidine) dihydrochloride (V‐50) was used as W_2_ phase for constructing the inner PEGDA shell. Deionized water containing 0.5% (w/v) Pluronic F‐127, 4% (w/v) chitosan, and 1.5% (w/v), with adjusted pH = 6.6, was used as W_4_ phase for constructing the outer chitosan shell. Photo‐initiator 2,2‐dimethoxy‐2‐phenylacetophenone (BDK) (0.5%, w/v) was added in the original O_3_ phase. TA (0.2%, w/v) was added in the original collection solution for crosslinking the chitosan. LR300 (0.1%, w/v) was added in the original O_1_ phase as model chemical molecule (Table S10, Supporting Information). The flow rates of O_1_, W_2_, O_3_, W_4_, and O_5_ for emulsion generation were 210, 420, 900, 2400, and 8500 µL h^−1^, respectively. After collected in the collection solution, the quadruple emulsions were converted into PEGDA@CS microcapsules under UV irradiation for 15 min in an ice‐water bath.

*Fabrication of Trojan‐Horse‐Like CS@PNIPAM Microcapsules*: For fabricating Trojan‐horse‐like CS@PNIPAM microcapsules, deionized water containing Pluronic F‐127 (0.5%, w/v), chitosan (4%, w/v), and HEC (1.5%, w/v), with adjusted pH = 6.6, was used as W_2_ phase for constructing the inner chitosan shell. Deionized water with monomer *N*‐isopropylacrylamide (NIPAM) (11.3%, w/v) (Sigma‐Aldrich), crosslinker *N*,*N*′‐methylene bisacrylamide (BIS) (0.77%, w/v), V‐50 (0.5%, w/v), Pluronic F‐127 (0.5%, w/v), and glycerol (5%, w/v) was used as W_4_ phase for constructing the outer PNIPAM shell. TA was added in the original O_1_ and O_3_ with contents of 2% (w/v) and 0.2% (w/v), respectively, for chitosan crosslinking. Photo‐initiator BDK (0.5%, w/v) was added in the original collection solution. LR300 (0.1%, w/v) was added in O_1_ phase as the model chemical molecule (Table S12, Supporting Information). The flow rates of O_1_, W_2_, O_3_, W_4_, and O_5_ for emulsion generation are 200, 440, 960, 2200, and 9000 µL h^−1^, respectively. After collected in the collection solution, the quadruple emulsions were converted into CS@PNIPAM microcapsules under UV irradiation for 15 min in an ice‐water bath.

*Morphological Characterization of the Trojan‐Horse‐Like Microcapsules*: The morphologies of the Trojan‐horse‐like microcapsules were studied by using CLSM (TCS SP5‐II, Leica) and SEM (G2 Pro, Phenom‐word BV). To better investigate the capsule‐in‐capsule structures, the morphologies of microcapsules, with oil cores removed by washing with isopropanol and water, were characterized by CLSM and SEM.

*Trojan‐Horse‐Like Microcapsules for Programmed Sequential Stimuli‐Responsive Release*: The programmed sequential stimuli‐responsive release behaviors of the Trojan‐horse‐like microcapsules were recorded by using CLSM. Samples in a home‐made transparent glass container were placed on a thermostatic stage system (TSA02i, Instec) mounted on the CLSM. The sequential release behaviors of CS@CS microcapsules were triggered by using buffer solution with pH = 2.5 (citric acid‐disodium hydrogen phosphate, 0.01 m) to provide an acid trigger. First, the samples in the glass container were placed on the thermostatic stage system at 25 °C for 30 min to reach a thermal equilibrium state. The buffer solution was also equilibrated at 25 °C in water bath. Then, the deionized water around the samples was replaced by the buffer solution rapidly and the sequential acid‐triggered burst release behaviors were recorded. Besides, the acid‐triggered shell decomposition behavior of CS@CS microcapsules without O_1_ and O_3_ phases was also studied by using CLSM to monitor the time‐dependent change of fluorescent intensity in the selected region of inner and outer chitosan shells. The sequential release of PEGDA@CS microcapsules was studied by using an acid trigger to achieve the first‐stage burst release of the inner PEGDA capsule for further diffusion‐based sustained release. Similarly, the buffer solution was used to provide the acid trigger. The sequential release of CS@PNIPAM microcapsules was studied by using a thermal trigger first, and then an acid trigger. The samples were equilibrated at 25 °C for 30 min at first, and then the temperature of deionized water in the containers was increased to 50 °C to trigger the first‐stage release. After that, the samples were cooled down to 25 °C, and the surrounding deionized water was rapidly replaced by the buffer solution to achieve a second‐stage release. Besides, the temperature‐dependent diameter changes of their outer PNIPAM shell in CS@PNIPAM microcapsules without O_1_ and O_3_ phases were also studied by using CLSM.

## Conflict of Interest

The authors declare no conflict of interest.

## Supporting information

Supplementary

Supplementary

Supplementary

Supplementary
