# Supplementary material for: Trojan‐Horse‐Like Stimuli‐Responsive Microcapsules
Source: Adv Sci (Weinh). 2018 Mar 13;5(6):1700960. doi: 10.1002/advs.201700960 (PMC6010793; doi:10.1002/advs.201700960)
Supplement: Supplementary file 1 — Supplementary [file ADVS-5-1700960-s002.pdf]

Copyright WILEY-VCH Verlag GmbH & Co. KGaA, 69469 Weinheim, Germany, 2018.

ADVANCED SCIENCE
Open Access

Supporting Information

for *Adv. Sci.,* DOI: 10.1002/advs.201700960

Trojan-Horse-Like Stimuli-Responsive Microcapsules

Chuan-Lin Mou, Wei Wang, Zhi-Lu Li, Xiao-Jie Ju, Rui Xie, Nan-Nan Deng, Jie Wei, Zhuang Liu, and Liang-Yin Chu**

Copyright WILEY-VCH Verlag GmbH & Co. KGaA, 69469 Weinheim, Germany, 2018.

Supporting Information

Trojan-Horse-like Stimuli-Responsive Microcapsules

Chuan-Lin Mou, Wei Wang, Zhi-Lu Li, Xiao-Jie Ju, Rui Xie, Nan-Nan Deng, Jie Wei, Zhuang Liu and Liang-Yin Chu**

Contents

Supplementary Figures S1-S3.

Supplementary Tables S1-S12.

Supplementary Movies S1-S3.

Supplementary Figures S1-S3.

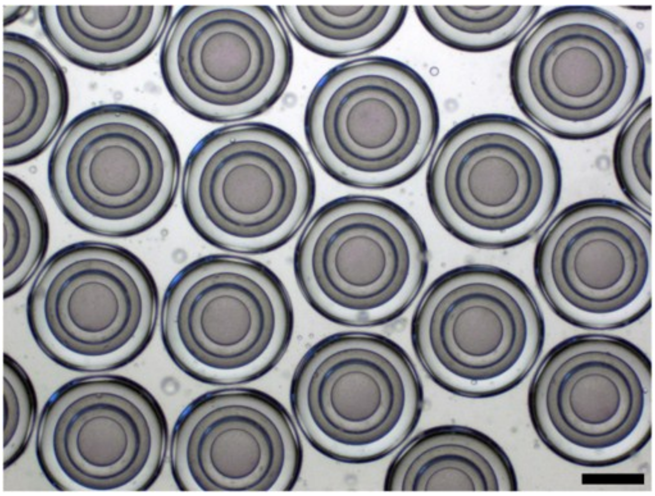

Figure S1. Optical micrograph of $O_1/W_2/O_3/W_4/O_5$ quadruple emulsions with recipe-IV at initial state. Scale bar is 200 μm.

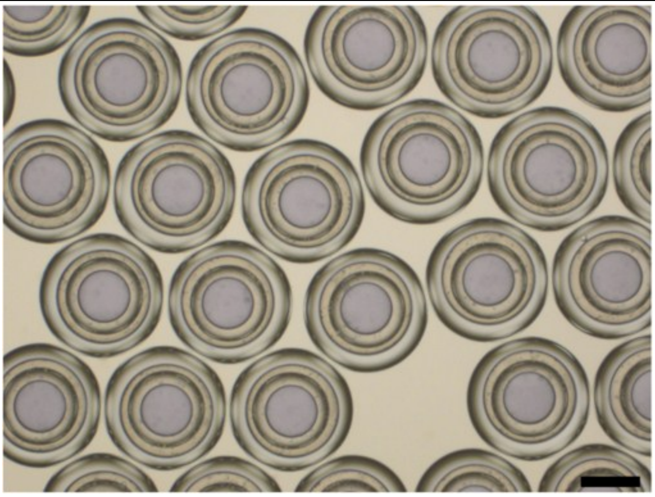

Figure S2. Optical micrographs of $O_1/W_2/O_3/W_4/O_5$ quadruple emulsions with recipe-V at initial state. Scale bar is 200 µm.

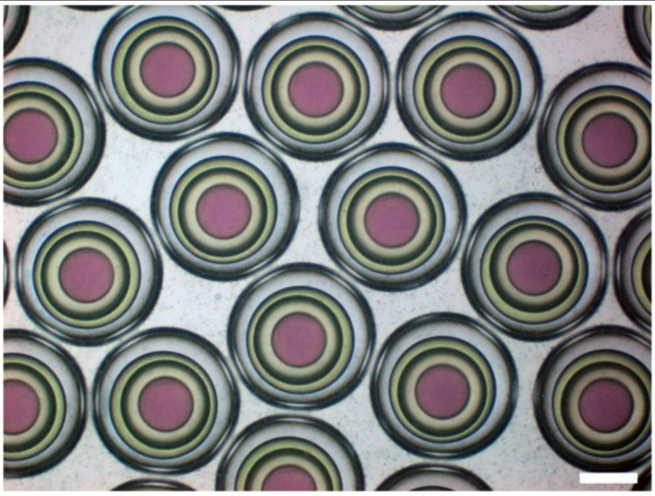

Figure S3. Optical micrographs of $O_1/W_2/O_3/W_4/O_5$ quadruple emulsions with recipe-VIII at initial state. Scale bar is 200 µm.

Supplementary Tables S1-S12.

Table S1. Recipe-I of $O_1/W_2/O_3/W_4/O_5$ quadruple emulsions and their densities and viscosities.

Phase	Composition	Density (g/mL)	Viscosity (mPa·s)
O_1	SO/BB (V_{SO}:V_{BB}=46:54) + PGPR (2%, w/v) + Sudan Black (0.1%, w/v)	1.025	20.47
W_2	H_2O + Pluronic F-127 (0.5%, w/v) + glycerin (10%, w/v)	1.024	1.168
O_3	SO/BB (V_{SO}:V_{BB}=46:54) + PGPR (4%, w/v) + LR 300 (0.1%, w/v)	1.025	21.82
W_4	H_2O + Pluronic F-127 (0.5%, w/v) + glycerin (10%, w/v)	1.024	1.168
O_5	SO + PGPR (5%, w/v)	0.917	63.46
Collection solution	SO + PGPR (5%, w/v)	0.917	63.46

Table S2. Recipe-II of $O_1/W_2/O_3/W_4/O_5$ quadruple emulsions and their densities and viscosities.

Phase	Composition	Density (g/mL)	Viscosity (mPa·s)
O_1	SO + PGPR (2%, w/v) + LR 300 (0.1%, w/v)	1.025	55.36
W_2	H_2O + Pluronic F-127 (0.5%, w/v) + glycerin (10%, w/v)	1.024	1.168
O_3	SO + PGPR (4%, w/v)	0.916	61.68
W_4	H_2O + Pluronic F-127 (0.5%, w/v) + glycerin (10%, w/v)	1.025	1.168
O_5	SO + PGPR (5%, w/v)	0.917	63.46
Collection solution	SO + PGPR (5%, w/v)	0.917	63.46

Table S3. Recipe-III of $O_1/W_2/O_3/W_4/O_5$ quadruple emulsions for fabricating CS@CS microcapsules and their densities and viscosities.

Phase	Composition	Density (g/mL)	Viscosity (mPa·s)
O_1	SO/BB (V_{SO}:V_{BB}=46:54) + PGPR (2%, w/v) + TA (2%, w/v) + Sudan Black (0.1%, w/v)	1.025	20.02
W_2	H_2O + Pluronic F-127 (0.5%, w/v) + chitosan (4%, w/v)	1.009	4.1
O_3	SO/BB (V_{SO}:V_{BB}=46:54) + PGPR (4%, w/v) + TA (0.2%, w/v) + LR 300 (0.1%, w/v)	1.029	22.07
W_4	H_2O + Pluronic F-127 (0.5%, w/v)+ chitosan (4%, w/v) + HEC (1.5%, w/v)	1.013	11.5
O_5	SO + PGPR (5%, w/v)	0.917	63.46
Collection solution	SO + PGPR (5%, w/v) + TA (0.2%, w/v)	0.917	63.46

Table S4. Interfacial tensions between phases of $O_1/W_2/O_3/W_4/O_5$ quadruple emulsions with Recipe-III.

Interface between phases	Interfacial tension (mN/m)
O_1/W_2	0.11
W_2/O_3	0.121
O_3/W_4	0.114
W_4/O_5	0.104

Table S5. Recipe-IV of $O_1/W_2/O_3/W_4/O_5$ quadruple emulsions for fabricating CS@CS microcapsules and their densities and viscosities.

Phase	Composition	Density (g/mL)	Viscosity (mPa·s)
O_1	SO/BB (V_{SO}:V_{BB}=46:54) + PGPR (2%, w/v) + TA (2%, w/v)	1.025	20.02
W_2	H_2O + Pluronic F-127 (0.5%, w/v)+ chitosan (4%, w/v) + HEC (1.5%, w/v)	1.013	11.5
O_3	SO/BB (V_{SO}:V_{BB}=46:54) + PGPR (4%, w/v) + TA (0.2%, w/v)	1.029	22.07
W_4	H_2O + Pluronic F-127 (0.5%, w/v) + chitosan (4%, w/v)	1.009	4.1
O_5	SO + PGPR (5%, w/v)	0.917	63.46
Collection solution	SO + PGPR (5%, w/v) + TA (0.2%, w/v)	0.917	63.46

Table S6. Interfacial tensions between phases of $O_1/W_2/O_3/W_4/O_5$ quadruple emulsions with Recipe-IV.

Interface between phases	Interfacial tension (mN/m)
O_1/W_2	0.091
W_2/O_3	0.114
O_3/W_4	0.121
W_4/O_5	0.109

Table S7. Recipe-V of $O_1/W_2/O_3/W_4/O_5$ quadruple emulsions for fabricating CS@CS microcapsules and their densities and viscosities.

Phase	Composition	Density (g/mL)	Viscosity (mPa·s)
O_1	SO/BB (V_{SO}:V_{BB}=46:54) + PGPR (2%, w/v) + TA (2%, w/v)	1.025	20.02
W_2	H_2O + Pluronic F-127 (0.5%, w/v)+ chitosan (4%, w/v) + HEC (1.5%, w/v)	1.013	11.5
O_3	SO + PGPR (4%, w/v) + TA (0.2%, w/v)	0.917	62.37
W_4	H_2O + Pluronic F-127 (0.5%, w/v) + chitosan (4%, w/v) + HEC (1.5%, w/v)	1.013	11.5
O_5	SO + PGPR (5%, w/v)	0.917	63.46
Collection solution	SO + PGPR (5%, w/v) + TA (0.2%, w/v)	0.917	63.46

Table S8. Interfacial tensions between phases of $O_1/W_2/O_3/W_4/O_5$ quadruple emulsions with Recipe-V.

Interface between phases	Interfacial tension (mN/m)
O_1/W_2	0.091
W_2/O_3	0.098
O_3/W_4	0.098
W_4/O_5	0.104

Table S9. Recipe-VI of $O_1/W_2/O_3/W_4/O_5$ quadruple emulsions for fabricating CS@CS microcapsules and their densities and viscosities.

Phase	Composition	Density (g/mL)	Viscosity (mPa·s)
O_1	SO/BB (V_{SO}:V_{BB}=46:54) + PGPR (2%, w/v) + TA (2%, w/v) + Sudan Black (0.1%, w/v)	1.025	20.02
W_2	H_2O + Pluronic F-127 (0.5%, w/v)+ chitosan (4%, w/v) + HEC (1.5%, w/v)	1.013	11.5
O_3	SO/BB (V_{SO}:V_{BB}=46:54) + PGPR (4%, w/v) + TA (0.2%, w/v) + LR300 (0.1%, w/v)	0.917	62.37
W_4	H_2O + Pluronic F-127 (0.5%, w/v)+ chitosan (4%, w/v) + HEC (1.5%, w/v)	1.013	11.5
O_5	SO + PGPR (5%, w/v)	0.917	63.46
Collection solution	SO + PGPR (5%, w/v) + TA (0.2%, w/v)	0.917	63.46

Table S10. Recipe-VII of $O_1/W_2/O_3/W_4/O_5$ quadruple emulsions for fabricating PEGDA@CS microcapsules.

Phase	Composition
O_1	SO/BB (V_{SO}:V_{BB}=46:54) + PGPR (2%, w/v) + LR300 (0.1%, w/v)
W_2	H_2O + PEGDA (10%, w/v) + Pluronic F-127 (0.5%, w/v) + V-50 (0.5%, w/v)
O_3	SO/BB (V_{SO}:V_{BB}=46:54) + PGPR (4%, w/v) + BDK (0.5%, w/v)
W_4	H_2O + Pluronic F-127 (0.5%, w/v)+ chitosan (4%, w/v) + HEC (1.5%, w/v)
O_5	SO + PGPR (5%, w/v)
Collection solution	SO + PGPR (5%, w/v) + TA (0.2%, w/v)

Table S11. Recipe-VIII of $O_1/W_2/O_3/W_4/O_5$ quadruple emulsions for fabricating CS@PNIPAM microcapsules and their densities and viscosities.

Phase	Composition	Density (g/mL)	Viscosity (mPa·s)
O_1	SO/BB (V_{SO}:V_{BB}=46:54) + PGPR (2%, w/v) + TA (2%, w/v) + LR300 (0.1%, w/v)	1.025	20.02
W_2	H_2O + Pluronic F-127 (0.5%, w/v)+ chitosan (4%, w/v)	1.009	4.1
O_3	SO + PGPR (4%, w/v) + TA (0.2%, w/v)	0.917	62.37
W_4	H_2O + NIPAM (11.3%, w/v) + Pluronic F-127 (0.5%, w/v) + glycerol (5%, w/v) + BIS (0.77%, w/v) + V-50 (0.5%, w/v)	0.996	1.68
O_5	SO + PGPR (5%, w/v)	0.917	63.46
Collection solution	SO + PGPR (5%, w/v) + BDK (0.5%, w/v)	0.917	63.46

Table S12. Recipe-IX of $O_1/W_2/O_3/W_4/O_5$ quadruple emulsions for fabricating CS@PNIPAM microcapsules and their densities and viscosities.

Phase	Composition	Density (g/mL)	Viscosity (mPa·s)
O_1	SO/BB (V_{SO}:V_{BB}=46:54) + PGPR (2%, w/v) + TA (2%, w/v) + LR300 (0.1%, w/v)	1.025	20.02
W_2	H_2O + Pluronic F-127 (0.5%, w/v)+ chitosan (4%, w/v) + HEC (1.5%, w/v)	1.013	11.5
O_3	SO + PGPR (4%, w/v) + TA (0.2%, w/v)	0.917	62.37
W_4	H_2O + NIPAM (11.3%, w/v)+ Pluronic F-127 (0.5%, w/v) + glycerol (5%, w/v) + BIS (0.77%, w/v) + V-50 (0.5%, w/v)	0.996	1.68
O_5	SO + PGPR (5%, w/v)	0.917	63.46
Collection solution	SO + PGPR (5%, w/v) + BDK (0.5%, w/v)	0.917	63.46

Supplementary Movies S1-S3.

Movie S1. Generation of $O_1/W_2/O_3/W_4/O_5$ quadruple emulsions via sequential emulsification in microfluidic device.

Movie S2. Chitosan@chitosan microcapsules for programmed sequential release. pH=6.5→2.5, T=25 ^{o}C.

Movie S3. Chitosan@PNIPAM microcapsules for burst release of the inner chitosan capsule. T=25 ^{o}C→50 ^{o}C, pH=6.5.
